# The economic burden of HIV/AIDS on individuals and households in Nepal: a quantitative study

**DOI:** 10.1186/s12913-017-1976-y

**Published:** 2017-01-24

**Authors:** Ak Narayan Poudel, David Newlands, Padam Simkhada

**Affiliations:** 1 International Public Health Researcher, Public Health Institute, Faculty of Education, Health and Community, Liverpool John Moores University, England, UK; 2 Institute for Global Health and Development, Queen Margret University, Edinburgh, UK; 3 International Public Health, Public Health Institute, Faculty of Education, Health and Community, Liverpool John Moores University, England, UK

**Keywords:** Coping strategies, Direct costs, Economic burden, HIV/AIDS, Nepal, Productivity costs

## Abstract

**Background:**

There have been only limited studies assessing the economic burden of HIV/AIDS in terms of direct costs, and there has been no published study related to productivity costs in Nepal. Therefore, this study explores in detail the economic burden of HIV/AIDS, including direct costs and productivity costs. This paper focuses on the direct costs of seeking treatment, productivity costs, and related factors affecting direct costs, and productivity costs.

**Methods:**

This study was a cross-sectional, quantitative study. The primary data were collected through a structured face-to-face survey from 415 people living with HIV/AIDS (PLHIV). The study was conducted in six representative treatment centres of six districts of Nepal. The data analysis regarding the economic burden (direct costs and productivity costs) was performed from the household’s perspective. Descriptive statistics have been used, and regression analyses were applied to examine the extent, nature and determinants of the burden of the disease, and its correlations.

**Results:**

Average total costs due to HIV/AIDS (the sum of average total direct and average productivity costs before adjustment for coping strategies) were Nepalese Rupees (NRs) 2233 per month (US$ 30.2/month), which was 28.5% of the sample households’ average monthly income. The average total direct costs for seeking HIV/AIDS treatment were NRs 1512 (US$ 20.4), and average productivity costs (before adjustment for coping strategies) were NRs 721 (US$ 9.7). The average monthly productivity losses (before adjustment for coping strategies) were 5.05 days per person. The major determinants for the direct costs were household income, occupation, health status of respondents, respondents accompanied or not, and study district. Health status of respondents, ethnicity, sexual orientation and study district were important determinants for productivity costs.

**Conclusions:**

The study concluded that HIV/AIDS has caused a significant economic burden for PLHIV and their families in Nepal. The study has a number of policy implications for different stakeholders. Provision of social support and income generating programmes to HIV-affected individuals and their families, and decentralising treatment services in each district seem to be viable solutions to reduce the economic burden of HIV-affected individuals and households.

**Electronic supplementary material:**

The online version of this article (doi:10.1186/s12913-017-1976-y) contains supplementary material, which is available to authorized users.

## Background

An estimated 36.9 million people were living with human immunodeficiency virus (HIV) or acquired immunodeficiency syndrome (AIDS) in the world. An estimated number of 1.2 million people died and 2 million people were newly infected by HIV/AIDS in a year [1]. Therefore, HIV/AIDS is one of the major burdens of disease globally.

After the introduction of multiple antiretroviral therapy (ART), HIV/AIDS became a chronic disease, and there is a need to provide long-term care and support for the ill person. HIV/AIDS is concentrated among adults of working age, unlike other diseases [2, 3]. Long-term illness due to HIV/AIDS demands a higher level of treatment costs for the HIV-affected households. Therefore, HIV/AIDS causes depletion of savings and productive assets, and increases the indebtedness of the HIV-affected households [4]. Moreover, the higher health care expenditure of the households reduces investment for nutritional food for the family members, investment for farming or business, and the education of the children. After the initiation of ART medicine, mortality rates have reduced, but still a considerable number of people (1.2 million) die due to HIV/AIDS every year [1]. Death during the working age of the victim is a major factor in the economic impact of HIV/AIDS [5]. The household level impact of HIV/AIDS includes direct costs, including medical and non-medical costs, and productivity costs such as loss of labour time, as a result of the morbidity of HIV positive household members, as well as time spent by others caring for them [6]. This evidence suggests that HIV/AIDS places significant economic pressure on households trying to pay for health care costs, and trying to make up for lost income.

If a member of a farming household is affected by HIV/AIDS at a young and productive age, the household either reduces the size of their farm due to the reduction in the number of farm (family) workers [7] or hires external labour to work on their farm to replace the ill family member and carer. Thus, there is a reduction in the number of family workers and an increment in the labour costs of the farm due to HIV/AIDS. HIV/AIDS cannot only kill the economically active population but will also destroy their experience; skills and knowledge built up over a period of years [2]. If a breadwinner dies, then the family struggles to cope, not only emotionally but also economically. Poverty increases if the household’s head dies and scarce resources are utilised during the period of ill health.

The financial coping mechanism for ill health plays an important role in the economic impact experienced by households [8]. Household income and savings, sale of assets, loans, borrowing and removing children from school are the major coping strategies used by HIV-affected households [4, 9–13]. Sale of productive assets like land, farm animals and farm equipment, directly affect the productivity of households within a short period, whilst loans, borrowing and removing children from school affect the productivity of households over a longer period. Moreover, the sale of property, like land and homes for the treatment of HIV/AIDS, may render HIV-affected household landless and/or homeless. The financial coping strategies may solve the short-term problems of the HIV-affected households but may also reduce the economic capacity of the households in the long run, and risk pushing them into further poverty.

Past studies about the economic impact of HIV/AIDS have reported that the disease-affected households generate relatively lower income than unaffected households [14, 15]: people living with HIV/AIDS (PLHIV) are often forced to leave their employment or business due to their illness [12]. The decline in health of the ill person causes further impact on the household [9, 15]. The need for life-long treatment due to the chronic nature of the disease can have life-long financial implications on such households [3].

Since the first case of HIV/AIDS was reported in 1988 in Nepal, the nature of the HIV epidemic has gradually moved from being a ‘low prevalence’ to a ‘concentrated epidemic’ [16] among injecting drug users, sex workers and migrant workers who travel between Nepal and India. Over 85% of HIV in Nepal is transmitted through sexual activities [17]. Out of 28 million of the Nepalese population [18], estimated 39,249 people are living with HIV/AIDS. However, the recently reported cases of HIV/AIDS is 26,702, which is 68% of the national estimates of HIV infection [17]. There is still a significant gap (32%) between officially estimated, and medically reported cases. The HIV prevalence among the adult population (age between 15–49 years) is officially estimated at 0.20% (adult male 0.28%, adult female 0.13%), which is a decreasing trend [16].

Lack of knowledge about health insurance or unavailability of such insurance for HIV/AIDS causes higher out-of-pocket payment for the disease-affected households in Nepal [19]. This evidence suggests that the economic impact of HIV/AIDS can be catastrophic to the HIV-affected households [20].

Poverty is an important factor in the propagation of HIV/AIDS. Poor people are more vulnerable for many reasons, including exposure to high-risk behaviours and poor access to health services [21]. HIV/AIDS and poverty are interconnected in a vicious circle [22, 23]. It is believed that HIV/AIDS causes poverty and worsens already existing poverty [24]. The effect of HIV/AIDS is especially severe on households, which are already in poverty [13, 25]. Moreover, there is no or very little social security (such as financial allowances) in developing countries such as Nepal. Therefore, HIV/AIDS has a severe economic impact on HIV-affected households in developing countries compared to developed countries.

Examining the overall spending in Nepal, we note that US$ 24.5 million was spent on HIV/AIDS. Of this total spending, 90% came from external sources and 10% from domestic sources [26]. Although there are supports from donors, NGOs (Non-Governmental Organisations)/INGOs (International Non-Governmental Organisations) and governments, HIV-affected households are still paying a significant amount of money for their HIV positive family member’s treatment and care - for example, for travel, diagnostic tests, medicines other than ART, lodging and food. Support from the government is limited to CD4 (Cluster of Differentiation four) test and ART medicines. A study report also highlighted travel cost as a major problem and reported that the HIV-affected households were facing financial constraints for HIV/AIDS treatment in Nepal [27].

There were few studies conducted on the direct costs to the households, but there were no studies which reported the productivity costs due to HIV/AIDS in Nepal. A review reported that there were no sufficient research on economic issues of HIV/AIDS in Nepal [19]. Only two studies assessed the direct costs of HIV/AIDS treatment [28, 29]. Nevertheless, these studies did not include all the components of treatment costs, and did not cover rural areas. There were no studies in Nepal reporting on actual productivity costs caused by HIV/AIDS, and determinants of direct and productivity costs [19]. Therefore, there has been a knowledge gap concerning these issues in the Nepalese context. The ‘cost of illness’ study presented below was conducted to explore in detail the economic burden of HIV/AIDS from the household’s perspective, in terms of direct costs and productivity costs, and to establish some determinants of these costs.

## Methods

This is a cross-sectional study that employed a quantitative approach to collect information. A face-to-face structured survey obtained information from the respondents. In 2011, there were 23 HIV treatment and care centres in Nepal. Out of these, six treatment and care centres from six districts were selected purposively, based on their coverage, location and accessibility. These centres were: BP Koirala Institute for Health Sciences, Dharan, Sunsari; Sukraraj Tropical and Infectious Diseases Hospital, Teku, Kathmandu; Bharatpur Hospital, Bharatpur, Chitwan; Rapti Sub-Regional Hospital, Dang; Bheri Zonal Hospital, Nepalgunj, Banke; and District Hospital, Silgadi, Doti.

### Sample size, sampling process and data collection

A total of 446 respondents were approached and 415 respondents agreed to participate in the survey (93% response rate). The source of information for the survey was PLHIV aged 18 or over and who had been diagnosed HIV positive more than a month prior to the survey. The PLHIV who came to the treatment centres for check-ups, counselling and medicines were accessed for the study. A purposive sampling method was employed to select the participants for easy recruitment and the active participation of the respondents in the research [30].

A survey questionnaire was used to collect data from the respondents (please ﻿see the Additional file 1). The questionnaire was prepared in the English and Nepali but face-to-face administration of the questionnaires was in Nepali. After completion of the surveys, the collected information was translated into English. Questionnaires were piloted and validated by conducting an initial study with 36 respondents. The main survey was conducted in 2011. The information from PLHIV was collected by the researcher with the help of local assistants. The researcher trained the local assistants in the field. The Interviewer Manual was prepared and given them to read first. The assistants were also observed, monitored and supported while they were conducting first few surveys to ensure the accuracy of the data, and consistency of interview style by the interviewers. The respondents who visited the HIV treatment and care centres were recruited for the study were asked to provide details of costs of HIV treatment to avoid possible under estimation of costs, since some patients also went to private clinics or hospitals for their treatment. The given information was crosschecked and edited immediately following the interview; and collected data were crosschecked afterwards by the researcher prior to the analysis.

This research was approved by Nepal Health Research Council (NHRC) (a government ethical approval body), the National Centres for AIDS and STD Control (NCASC), and the University of Aberdeen, UK. Informed consent was taken from the respondents before starting the survey. Anonymity and confidentiality were maintained throughout the research process from the data collection to the report dissemination phase. The collected information was solely used for the purpose of the study and kept confidential. No cash payment was made to the participants, in order to avoid ‘bias’ in the over-eager volunteering of information simply to please the researcher. This research has fully considered the ethical procedures at all stages of its research processes.

### Data analysis

Data obtained from the survey was entered into the SPSS version 20 spreadsheet, and after that coded and cleaned for analysis purposes. Data analysis was done using descriptive statistics - mainly means, standard deviation, frequencies, and percentages; and regression analyses. Measurement methods for direct costs, productivity costs and total costs are described below.

#### Measurement of average total direct cost

Direct costs in this study was measured from the household’s perspective that means household was the payer. Average total direct costs for HIV/AIDS treatment were measured by combining all the average out-of-pocket medical and non-medical costs for HIV infected individuals, as well as costs for accompanying person/s. These included costs of doctors, diagnostic tests, clinic or hospital charges, travel, food, lodging and other items at the time of treatment. Direct costs incurred for accompanying person/s attending for diagnosis and/or treatment were included in the measure of average total direct costs. In Nepal, PLHIV under ART medicine need to visit a HIV/AIDS treatment and counselling centre every month for treatment, routine check-up and counselling services. In addition, while conducting the survey, it was found that PLHIV who were not under ART also visited clinics, hospitals or treatment centres every month to see doctors to check their general health, and treat non-HIV major and minor illnesses (which nevertheless, may have been indirectly caused by immune system deficiency). Therefore, the direct cost for the last visit to the treatment centre was taken to be equivalent to the monthly direct costs due to HIV/AIDS in this study.

#### Measurement of average productivity cost

In this study, productivity cost was defined as the inability to carryout normal daily activities (paid and or unpaid work), and their valuation. Normal daily activities were defined as formal and non-formal work carried out by individuals in rural and urban settings.

To calculate the productivity losses, the inability of PLHIV was divided into absenteeism and presenteeism. Complete inability to carry out normal daily activities due to illness was defined as ‘absenteeism’, and ‘presenteeism’ was defined as reduced work efficiency due to ill health while still working. The sum of absenteeism and presenteeism was termed as ‘productivity losses’ and their monetary valuation was termed as ‘productivity cost’ in this study. To calculate the productivity losses due to presenteeism, two aspects were taken into account: the period during which losses were experienced and the extent to which work efficiencies were affected. To calculate the respondent’s days lost due to illness, respondents were asked whether they were completely unable to work in the last 2 months (60 days) or not. A visual analogue scale (VAS) ranging from blocks zero (0) to five (5) was used in asking individuals about work efficiency when they were ill but still working. Block zero meant ‘unable to work at all’, block one meant ‘one out of five efficiency’, and so on. Block five (5) meant illness did not affect work efficiency at all. Therefore, respondents would never answer block one or block five if they worked in a state of ill-health, because block zero means absenteeism and block five meant ‘not sick at all’. To calculate the days lost due to presenteeism, the total days worked in a state of poor health was multiplied by the inefficiency of the work during ill health.

The days recovered were also calculated in the same way by using a VAS as presenteeism. Firstly, respondents were asked whether they got help or not from others when they were ill. If they said ‘no’, then the days recovered would be zero (0). If they said ‘yes’, then they were asked on how many days they got help. To operationalise this, they were asked on a VAS with blocks of zero to five, how much work was completed by others helping. To calculate the days recovered from other’s help, the days helped by others were multiplied by the amount of work done by other’s help. Although respondents were asked the total days lost due to HIV/AIDS (absenteeism and presenteeism) in the last 2-month period, the total days lost were divided into two, to get monthly days lost due to HIV/AIDS. Thus, in this study, total days lost due to HIV/AIDS before adjustment of coping strategies (sum of absenteeism and presenteeism), total days recovered by other’s help, and days lost after adjustment by coping strategies were calculated.

Valuation of productivity losses in this study was done by using per capita gross domestic product (GDP) of Nepal (NRs 142.8/day, according to World Bank for 2011). Valuation using per capita GDP was preferred, because this approach values time loss of rich or poor people by an average for the whole society: it is not appropriate to value rich people’s time at a high rate and poor people’s time at low rate. It is specifically true for HIV/AIDS which unlike other diseases like malaria is not a disease of rich or poor; it affects both rich and poor alike. Likewise, productivity costs ‘before coping strategies’, is preferred rather than ‘after coping strategies’, since coping strategies do not make productivity costs disappear. As the name implies, these strategies simply allow people to cope better. Therefore, productivity costs before coping strategies, were valued by using per capita GDP, and was preferred over productivity costs after coping strategies.

The total costs in this study were calculated by summing the average total direct costs to the household, and the productivity costs in a monthly period.

#### Regression analysis

In this study, regression analyses were conducted to find the important predictor variables for treatment and access costs for PLHIV (as the direct costs), and productivity costs before coping strategies. Regression analyses for total direct costs to the household were not conducted because PLHIV who took an accompanying person obviously need to pay higher costs because of the additional costs required for the accompanying person/s. This might overshadow the impact of other important variables like the health status, and the income level of the PLHIV. Therefore, regression analyses for treatment and access costs in the place of total direct costs were used.

To conduct the regression analyses for treatment and access costs and productivity costs, the predictor variables which were significant to the relevant outcome variables in the descriptive analyses, were taken into consideration. Occupation, ethnicity, CD4 level, self-reported health status, PLHIV accompanied or not, household income and study district were found to be statistically significant variables for the direct costs in the descriptive statistics. Likewise, education, occupation, ethnicity, CD4 level, self-reported health status, PLHIV accompanied or not, and study district were found significant variables for productivity costs in descriptive statistics. As the health status was measured in two forms: self-reported health status and CD4 level, one regression with self-reported health status and another regression with CD4 level were conducted separately along with other significantly contributing variables. Likewise, household income was also presented in two forms: as a continuous variable, and in income quintiles; one regression with income (as a continuous variable) and another regression with income quintiles were conducted separately along with other significantly contributing variables. Thus, four different types of regression analyses for treatment and access costs and three regression analyses for productivity costs were conducted before conducting the tests (here test regression analysis means the analysis which was conducted to test the significant of other extra variables which did not show up as significant in the descriptive analyses for the outcome variable); and then, final regression analyses. After that, a number of separate regression analyses were conducted to check if the other variables which did not contribute significantly in the descriptive analyses did emerge significantly as synergistic, or interactive variables. After checking all results, a final list of significantly contributing predictor variables was prepared and used for the final, reduced form of regression analysis.

Before conducting the regression analyses, normality, linearity, multicolinearity, and outliers among the variables were checked. Normality and linearity were checked by using histogram and normal probability plot, multicolinearity was checked by using Pearson correlation, and outliers were checked by using Mahalanobis and Cook’s distance matrices. To correct the skewed data, log transformation (log10) was made for treatment and access costs, productivity costs (before coping strategies) and household income. All other variables which were either interval or nominal in nature, were rescored as binary variables. Examples of potential predictors which were changed into binary or dummy variables were: study districts, education of PLHIV, occupation of PLHIV, ethnicity of PLHIV, CD4 level of PLHIV, self-reported health status of PLHIV, and PLHIV accompanied with other.

As our outcome variables were log transferred and some of the predictor variables were also log transferred, the regression equation was:

1 $$ LogY=\alpha +{b}_{1\times }{X}_1+{b}_{2\times }log{X}_2+{b}_{3\times }{X}_3+\dots \dots \dots \dots ..+{b}_{k\times }{X}_k+e------- $$

Where,

*Log Y* = outcome variable (log transferred)

*X*_*1,*_ *X*_*2,*_ *X*_*3*, and_ *X*_*k*_ are predictor variables

α represents regression constant or intercept

*b*_*1,*_ *b*_*2,*_ *b*_*3*,_ and *b*_*k*_ are the unstandardized regression coefficients, where k represents the number of predictor variables, and e is the error.

In the above regression equation, predictor variable *X*_*2*_ is log transferred.

## Results

### Basic information on respondents

Out of 415 respondents surveyed, 50.6% were male and 93.3% were aged 18 to 49 years (mean 36 years). Almost 64% of respondents were from rural areas and 66% were literate (informal to higher level of education).

More than 25% of respondents were farmers, 45.8% were Brahmin/Chhetri and 94.5% were heterosexual. More than 67% of respondents were married; 79.5% were taking ART, and 46.5% were with CD4 level between 200/mm^3^ to 400/mm^3^. Around 58% of respondents had self-reported good health status, and 29.2% of respondents were accompanied by a family member, friend or relative while visiting the treatment centre. Although the insurance system in Nepal does not cover HIV positive people, five out of 415 respondents reported that they had health insurance, but never used insurance support for their treatment (Table 1).

**Table 1:** Socio-economic characteristics of the survey respondents

Characteristics	Frequency (*N*)	Percentage (%)
Study Districts (Treatment centres)		
Sunsari	60	14.5
Kathmandu	105	25.3
Chitwan	70	16.9
Dang	60	14.5
Banke	60	14.5
Doti	60	14.5
Gender		
Male	210	50.6
Female	205	49.4
Age Group of Respondents		
18 to 49 years	387	93.3
≥50 years	28	6.7
Location of the Respondents		
Rural	265	63.9
Urban	150	36.1
Educational Status		
Illiterate	141	34.0
Primary/informal class (can read & write)	146	35.2
Secondary/under SLC^a^	89	21.4
Above SLC	39	9.4
Main Occupation		
Employed/having job	97	23.4
Business/self employed	62	14.9
Agriculture	107	25.8
Household work	45	10.8
Unemployed	50	12.0
Wage labour	28	6.7
Other (student/retired/handicapped or very weak)	26	6.3
Ethnicity^b^		
Brahmin/Chhetri	190	45.8
Newar/Gurung/Thakali	38	9.2
Other Ethnic Groups	109	26.3
Dalits	57	13.7
Madeshi Tribes	21	5.1
Sexual Orientation		
Heterosexual	392	94.5
Lesbian/Gay/Bisexual/Transgender	23	5.5
Marital Status		
Married	280	67.5
Widow/widower	100	24.1
Unmarried/Divorced/Separated	35	8.4
Having ART or Not		
Having ART	330	79.5
Not Having ART	85	20.5
Current CD4 Level		
>400/mm3	113	27.2
200–400/mm3	193	46.5
<200/mm3	109	26.3
Current Health Status		
Good	243	58.6
Medium	138	33.3
Poor	34	8.2
Respondents with accompanying person		
Yes	121	29.2
No	294	70.8
Having Health Insurance		
Yes	5	1.2
No	410	98.8

^a^SLC means School Leaving Certificate, it is the same as a class ten pass. The students who pass class ten get this certificate which is required to get admission to college level

^b^In this study, caste systems are defined as ethnicity. There are five major castes in Nepal. Brahmin/Chhetri is the highest caste followed by ethnic groups. Ethnic groups are also divided into two: Newar/Gurung/Thakali as the well-off ethnic castes and other ethnic groups who are less well-off than Newar/Gururng/Thakali. Dalit are the most marginalised caste in Nepal and Madeshi Tribes are ethnic minorities who lives in Terai part of Nepal

### Average direct cost for HIV/AIDS treatment

It was found that the average direct costs to the HIV-affected household at the last visit to the treatment centre (including accompanying person) was NRs 1512 (US$ 20.4), which was 19.3% of the average household income (NRs 7837). Average treatment costs for HIV/AIDS on the last visit was NRs 922 (US$ 12.5), which was 61% of the average direct costs for the last visit. The highest treatment costs was accounted for by the cost for diagnostic tests (NRs 486.7, or US$ 6.6) (32.2%) followed by cost of medicine (NRs 313 (US$ 4.2) (20.7%). Average access costs for PLHIV on the last visit were found to be NRs 445.4 (US$ 6), which is 29.4% of average direct costs for the treatment on the last visit. Among the access costs, transportation costs accounted 18.5% of the average direct costs. Thus, total treatment and access costs for PLHIV on the last visit was NRs 1367.5 (US$ 18.5), which is 90.4% of the average direct costs for the last visit. The costs for accompanying person/s accounted for 9.6% of the average direct costs (NRs 144.5) (Table 2).

**Table 2:** Direct costs for the HIV/AIDS treatment at the last visit to the treatment centre

Cost categories	Average costs in NRs (Std. Deviation)	Percent of total direct costs (%)
Cost of doctors	63.9 (126.3)	4.2
Cost of diagnosis or test	486.7 (720.0)	32.2
Cost of medicine	313.0 (627.5)	20.7
Other medical costs	58.5 (181.3)	3.9
Total treatment costs	922.1 (1324.2)	61
Cost of transportation	279.0 (365.5)	18.5
Food cost of patient	106.8 (232.0)	7.1
Lodging cost and other access costs	59.6 (209.2)	3.9
Total access costs	445.4 (630.2)	29.4
Treatment and Access Costs	1367.5 (1564.4)	90.4
Cost for accompanying person	144.5 (419.4)	9.6
Total direct costs	1512 (1813.2)	100

While looking at the total direct costs of treatment per visit based on CD4 counts, significantly higher costs were experienced by the PLHIV who had CD4 counts equal to or less than 200/mm^3^ (NRs 2051.7) compared to the PLHIV who had CD4 counts more than 200/mm^3^ (NRs 1319.5) *(p* < 0.001).

### Average productivity cost due to HIV/AIDS

The study found that the average number of days on which a PLHIV was completely unable to carry out normal daily activities (absenteeism) in a month was 3.15, and average days lost while present at work in a state of poor health with reduced efficiency (presenteeism) in a monthly period were 1.9. Therefore, average productivity loss before adjustment by coping strategies in a monthly period was 5.05 days. Average total days recovered by others’ help was 1.45. After adjustment by coping strategies (i.e. subtracting days recovered by others help from the total days lost), average total days lost due to HIV/AIDS in a monthly period was 3.6. Here, coping strategies indicate the support from other people like family members, relatives, neighbours or friends who help the PLHIV for their care and any kind of work including household work.

Average productivity costs were calculated by multiplying average days lost due to HIV/AIDS in a monthly period with the per capita GDP of the country in 2011. Through valuing productivity losses before adjustment of coping strategies, and after adjustment of coping strategies, two figures of productivity costs were obtained. Thus, average productivity costs due to HIV/AIDS in a monthly period before and after adjustment by coping strategies were NRs 721 (US$ 9.7) and NRs 514 (US$ 6.9) respectively.

The proportion of productivity costs valued by per capita GDP before adjustment of coping strategies was 9.2%, and after adjustment for coping strategies was 6.6% of the average household income. While looking at the productivity costs before adjustment of coping strategies based on CD4 counts, the higher proportion was accounted for by PLHIV who had CD4 counts equal or less than 200/mm^3^ (NRs 1157.2/month) than the PLHIV who had CD4 counts more than 200/mm^3^ (NRs 565.7/month) (*p* < 0.001) (Table 3).

**Table 3:** Average productivity losses and costs in a monthly period due to HIV/AIDS among PLHA

Items	Average days lost (Std. Dev)	Valuation by using per capita GDP in 2011 (NRs 142.8/day) (World Bank data) (Average Productivity Costs)
Absenteeism (days completely unable to work) (A)	3.15 (6.96)	449.7
Presenteeism (days worked in poor health x reduced efficiency) (P)	1.9 (3.22)	271.3
Productivity loss before adjustment of coping strategies (A + P)	5.05 (7.62)	721
Days recovered by other’s help (total days help received x amount of work done) (DROH)	1.45 (2.11)	207
Productivity loss after adjustment of coping strategies [(A + P)–DROH)]	3.6 (6.03)	514

### Average total costs due to HIV/AIDS (direct costs and productivity costs)

Average total costs were calculated by adding the average total direct costs to the household due to HIV/AIDS at the last visit to the treatment centre with the average productivity costs due to HIV/AIDS in a monthly period. Average total costs due to HIV/AIDS for a month’s period were calculated by summing the average direct costs, and the average productivity costs due to HIV/AIDS in a monthly period.

Thus, average total costs due to HIV/AIDS before coping strategies by using the per capita GDP for valuation was NRs 2233 (US$ 30.2), which was 28.5% of the average household monthly income. The proportion of average total direct costs was 67.7% of the total costs of the HIV/AIDS (NRs 1512 of NRs 2233), and the proportion of average productivity costs to the average total costs was 32.3% (NRs 721 of NRs 2233).

### Determinants for treatment and access costs

The final reduced form of regression analysis (Table 4) showed that household income (log), the health status of PLHIV (CD4 level), occupation, PLHIV accompanied or not, and study district were the significant predictor variables. Specifically, household income (log) (*p* < 0.001), CD4 level 200–400/mm^3^ (*p* < 0.01), CD4 level <200/mm^3^ (*p* < 0.001), Kathmandu district (*p* <0.001) and PLHIV accompanied by others (*p* < 0.001) contributed positively to the treatment and access costs, but ‘household work’ as an occupation (*p* < 0.05) contributed negatively.

**Table 4:** Linear regression analysis for treatment and access costs (log)

*Final regression analysis reduced form*		
*Predictor variables*	*b*	*SE*
*Kathmandu*	.221***	.057
*Household work*	-.157*	.078
*CD4 level 200–400/mm* ^*3*^	.185**	.059
*CD4 level < 200/mm* ^*3*^	.265***	.067
*Household income (log)*	.307 ***	.076
*PLHA accompanied by others*	.154**	.054
*Constant*	1.473***	.292
*No. of observations*	410	
*R-square*	0.136	
*Adjusted R-squire*	0.123	
*p value*	0.000	

b means coefficient and SE means standard error, *p < 0.05, **p < 0.01, ***p < 0.001

The final regression analysis for the treatment and access costs (log) showed that the expected increase in treatment and access costs was 3%, with an increment of household income of 10%, holding other variables constant. The expected increase in treatment and access costs from CD4 level >400/mm^3^ to 200–400/mm^3^ was 20.3%, holding other variables constant. The expected increase in treatment and access costs from CD4 level >400/mm3 to <200/mm3 was 30.3%, holding other variables constant. The expected increase in treatment and access costs from the Doti district to the Kathmandu district was 24.7%, holding other variables constant. The expected increase in treatment and access costs from PLHIV unaccompanied to PLHIV accompanied by others was 16.6%, holding other variables constant. The expected decrease in treatment and access costs from agricultural occupation to household work was 17%, holding other variables constant (Table 4).

The regression analyses confirm that household income, health status of PLHIV (CD4 level), occupation of PLHIV, PLHIV accompanied by others, and study district were the important predictor variables of the treatment and access costs. Thus, the treatment and access costs were highest for PLHIV having the highest household income, PLHIV having CD4 level <200/mm^3^, PLHIV coming to Kathmandu district for treatment and PLHIV accompanied by others. The treatment and access costs were lowest among the PLHIV with household work occupation.

### Determinants for productivity costs

Final reduced form of regression analysis (Table 5) showed that health status, ethnicity, sexual orientation, and study district were important predictor variables for productivity costs. Specifically, self-reported medium health status (*p* < 0.001), self-reported poor health status (*p* < 0.001), Dalit (*p <* 0.01), LGBT (lesbian, gay, bisexual and transgender) (*p* < 0.05), Sunsari district (*p* < 0.001), Chitwan district (*p* < 0.001), Dang district (*p* < 0.001), Banke district (*p* < 0.001), were positively contributing to the productivity costs.

**Table 5:** Linear regression analysis for productivity costs before coping strategies (log)

*Final regression analysis reduced form*			
*Predictor variables*	*b*		*SE*
*Sunsari*	.405***		.070
*Chitwan*	.533***		.066
*Dang*	.357***		.072
*Banke*	.321***		.070
*Dalit*	.254***		.066
*Self-reported medium health status*	.253***		.051
*Self-reported poor health status*	.824***		.084
*LGBT*	.217*		.099
*Constant*	2.246		.046
*No. of observations*		314	
*R-square*		0.413	
*Adjusted R-square*		0.397	
*p value*		0.000	

b means coefficient and SE means standard error, *p < 0.05, **p < 0.01, ***p < 0.001

The final regression analysis for the productivity costs (log) shows that the expected increase in productivity costs from the Brahmin/Chhetri to the Dalit caste was almost 29%, holding other variables constant. The expected increase in productivity costs from self-reported good health status to self-reported medium health status was 29.8%, holding other variables constant. The expected increase in productivity costs from self-reported good health status to self-reported poor health status was 128%, holding other variables constant. The expected increase in productivity costs from heterosexual PLHIV to LGBT (lesbian, gay, bisexual and transgender) PLHIV was 24.2%, holding other variables constant. The expected increase in productivity costs from the Doti district to the Sunsari district was almost 50%, holding other variables constant. The expected increase in productivity costs from the Doti district to the Chitwan district was 70.4%, holding other variables constant. The expected increase in productivity costs from the Doti district to the Dang district was almost 43%, holding other variables constant. The expected increase in productivity costs from the Doti district to the Banke district was almost 38%, holding other variables constant (Table 5).

Thus, productivity costs were highest among PLHIV in the Chitwan district, Dalit caste, self-reported poor health status and among LGBT.

## Discussion

Average total direct costs to the HIV-affected household (NRs 1512 or US$20.4 per visit) for HIV/AIDS treatment in our study were higher than other studies on HIV/AIDS conducted in Nepal [28, 29]. Higher direct costs in our study were due to inclusion of all costs components and various geographical locations, availability of better but expensive diagnostic and treatment facilities in the private and government hospitals in recent years, and higher awareness levels among PLHIV than before. The average total direct costs for HIV treatment in our study were higher than reported for tuberculosis, water borne diseases and malaria treatment in Nepal [31–33]. However, we cannot compare other studies in Nepal with costs of kala-azar (visceral leishmaniosis or VL) [8, 34], and hepatitis E treatment [35] because of methodological differences in calculation of the costs.

Our study findings show that the highest proportion of direct costs was accounted for by diagnostic tests (32.2%). There is difference between costs of treatment (i.e. doctor’s fee, diagnostic tests, medicines etc.) in government hospitals and private clinics or hospital. The treatment costs increase significantly if the PLHIV visits to the private clinics or hospitals. Although the PLHIV suspect that they might have HIV, they do not visit the hospital or clinic until they are very ill because of possible discrimination from families, relatives and society. This further helps to increase the costs of treatment. Access costs accounted for the second highest proportion (29.4%) of direct costs. There are no HIV/AIDS treatment and care services in every district of Nepal, meaning some PLHIV need to travel farther resulting into higher access costs. A previous study also mentioned the distance from health care facilities as a main problem in getting HIV/AIDS treatment services in Nepal [27]. Moreover, there are no insurance facilities for PLWA in Nepal, which forces individuals to pay out-of-pocket for their treatment. This puts the majority of HIV-affected families at risk of falling into ‘the poverty trap’.

In our study, HIV-affected households spent more than a five times higher proportion of household income for HIV/AIDS (19.3%) than reported by the government of Nepal for general health care (3.3% of average household income) [36], even though nearly half of the sample households (47.2%) were living in poverty, compared to the general population figure (25%). This evidence shows that HIV-affected households pay a considerably higher proportion of their income on healthcare compared to the general population.

While comparing our study findings with similar studies conducted in other countries, the average total direct costs of HIV/AIDS treatment in our study is similar to the total median costs in South India [4] and comparable to Vietnam [20] and Malaysia [37]. However, a study in Chad reported the average total costs more than four times higher than those found in our study [13]. The lower average direct costs in our study compared with the findings in Chad were mainly due to inclusion of both AIDS and non-AIDS respondents. However, a study conducted in Benin reported almost half the costs to access the package of care for ART therapy, than those from our own study findings [38]. The higher direct costs in our study than the study in Benin may be due to methodological differences, as they assessed the costs only to get ART medicine, unlike in our study.

Average total productivity losses (absenteeism and presenteeism) due to HIV/AIDS before adjustment for coping strategies in Nepal was found to be very high in a monthly period (5.05 days). Per month absenteeism (days completely unable to carry out normal daily activities) was 3.15 days and presenteeism (days lost due to reduced working efficiency because of poor health) was 1.9 days. Productivity costs before adjustment for coping strategies by using per capita GDP for valuation was NRs 721 (US$9.7) per month, which was 9.2% of the average monthly household income. The proportion of productivity costs to total costs (sum of direct costs and productivity costs) before adjustment of coping strategies was 32.3%, when doing a valuation using per capita GDP (NRs 721 of NRs 2233).

There have been no studies conducted in Nepal, which measured productivity losses due to HIV/AIDS. The findings of the studies on kala-azar (VL) [34, 39], hepatitis E [35] and malaria [33] could not be compared because of methodological differences in calculating days lost, since these studies calculated costs on a per episode basis.

We note that studies related to productivity losses due to HIV/AIDS in other countries, studies in India [4] and Malaysia [40] reported lower productivity losses than were found in our study. The productivity losses among AIDS patients reported in Chad [13] is comparable to the productivity losses among PLHIV with a self-reported poor health status in our study. The higher productivity losses in our study might be for two reasons. Firstly, PLHIV in Nepal do not have access to a balanced diet required to keep them healthy, due to their chronic poverty. This may weaken their immune system, making them more vulnerable to opportunistic infections. Secondly, we also included presenteeism in productivity losses unlike other studies [4, 40].

Average total costs (sum of average total direct costs and average productivity costs before adjustment of coping strategies, by using per capita GDP for valuation) due to HIV/AIDS in Nepal was NRs 2233 (US$ 30.2), which was 28.5% of an average household monthly income. There were no studies in Nepal assessing the per month average total cost of HIV/AIDS to compare to our study. The average total costs due to HIV/AIDS in our study are considerably higher compared to other diseases in Nepal, because of its chronic nature. Other diseases like tuberculosis, kala-azar (VL), hepatitis E, and malaria can be healed through treatment; therefore, HIV/AIDS causes a higher and long-term economic burden on the HIV-affected households than is caused by the above-mentioned diseases.

Looking for studies similar to our own, we found few that had developed a complex methodology of measuring economic costs that could be compared with our results. One study in Chad reported the total cost for AIDS care to be considerably higher than our study finding. However, they studied only AIDS patients (a more serious stage of HIV/AIDS), and included funeral costs in their study. A study in Spain reported total costs of care for asymptomatic HIV, symptomatic HIV and AIDS patients to be relatively higher than the total costs found in our study [41].

In our study, treatment and access costs (in terms of direct costs) were significantly determined by the health status of respondents (CD4 level 200–400/mm^3^ – *p* < 0.01, CD4 level <200/mm^3^ – *p* < 0.001), household income (*p* < 0.001), occupation of respondents (*p* < 0.05), respondents accompanied by others (*p* < 0.01) and study district (*p* < 0.001).

As mentioned above, the CD4 level as a measure of the health status of respondents was found to be one of the significant predictor variables for the treatment and access costs. The respondents with lower CD4 level had to pay higher treatment and access costs compared to respondents with a higher CD4 level. This finding is supported by the studies conducted in in India [4], Italy [42] and Spain [41]. Another significant predictor variable for the treatment and access costs was household income. Respondents having a higher household income paid higher treatment and access costs. This finding was supported by a study in India [4]. Occupation was found to be another significant predictor variable for the treatment and access costs. The respondents with household work as their occupation had paid significantly lower treatment and access costs than the respondents with agriculture (farming) as their occupation. The descriptive analysis shows that respondents with an agriculture occupation paid higher access costs than respondents with household work as their occupation. This evidence suggests that respondents with an agriculture occupation travel further for their treatment, resulting in higher treatment and access costs. Study district and PLHIV accompanied by others were found to be other significant predicator variables for treatment and access costs, the reason being that respondents who need to go far for their treatment generally took an accompanying person. Likewise, respondents travel farther for the better treatment facilities which are not available in their local area. Therefore, longer travel distance and use of better treatment facilities increased the treatment and access costs for respondents who took an accompanying person them.

The health status of respondents was a significant predictor variable for productivity costs. This finding is also supported by studies from India and Switzerland [4, 43]. Ethnicity was found to be another significant predictor variable for the productivity costs. Dalit (lower class) respondents had higher productivity costs than Brahmin/Chhetri respondents. The reason may be their poor economic status, as they cannot afford healthy diet, or timely treatment, which require them to fight against infections. Sexual orientation was also found to be a significant predictor variable for productivity costs. Productivity costs were higher for LGBT (lesbian, gay, bisexual and transgender). The reason may be related to the risky behaviour (e.g. - injecting drugs) adopted by the LGBT respondents. It has been reported that substance abuse is seven times higher among LGBTs than heterosexuals [44]. Use of serious drugs may be one of the most important factors to increase productivity losses among LGBTs. This argument is indirectly supported by a study in Switzerland, which reported intravenous drug use as the important determinant of productivity costs among PLHIV [43]. Study district was another important predictor variable for productivity costs. The variation in productivity losses by study site has been supported by other studies [39, 45].

Surprisingly, income was not a significant predictor variable for the productivity costs. It was found that productivity losses between the poorest respondents (first income quintile) and the richest respondents (fifth income quintile) were almost the same (5.5 days vs 5 days). The poorest PLHIV may be sicker than the richest PLHIV due to the unaffordability of treatment in time and lack of a nutritious diet. However, they have to work every day for their own and their family’s livelihood, although they are sick [46]. Perhaps, the richest respondents may be less sick than the poorest respondents (due to affordability of treatment in time or better diet), or they can afford to take time off to be sick.

This is the first study of its kind in Nepal. The sample of the study is also representative of Nepal’s population. This is because it has a relatively large sample size (>400), and was conducted in six representative districts of Nepal, which cover five development regions, east to west, and hilly and mountainous to Terai (the plains) regions of the country. In addition, the study included both rural and urban areas unlike previous studies, which were focussed only in urban areas. While calculating costs of illness, previous studies had excluded some important cost components like ‘costs for accompanying person’ in attempting to calculate direct costs, and cost of presenteeism in calculating productivity costs. Our study has included all components of the direct costs and productivity costs.

This study was conducted in government operated HIV/AIDS treatment centres. Therefore, it excluded those PLHIV who did not visit the treatment centre during the period of the study. Due to the limitations of time and resources, control groups (e.g. - HIV negative people, or people with other disease profiles) were not accessed. Cross-sectional studies only capture information from respondents at a certain point of time when data collection is carried out. Therefore, information collected in cross-sectional studies may not be as robust as information collected from longitudinal studies because variations in study subjects may be affected by weather, seasons and time of study. Most of the data in the study were derived from reporting of respondents who needed to recall their past. Therefore, there might be a possibility of recall bias by respondents about the given information and uncertainty about the robustness of the data. Moreover, the data in the study was collected in 2011. There might be changes in costs and burden to the HIV-affected households now due to changes in socioeconomic factors affecting the burden over the period.

## Conclusions

Although the doctor’s fees and medicine costs in the government hospitals in Nepal are minimal, the HIV-affected households are still paying a considerable amount of money (relative to their incomes) for diagnostic tests, transportation, food and lodging. Therefore, the Government of Nepal should make a policy for the affordable and accessible treatment of HIV/AIDS for everyone. It is recommended that the economic burden of HIV/AIDS could be reduced by decentralising HIV/AIDS related services to district level and putting in place more comprehensive service delivery for HIV/AIDS care, support and treatment.

Subsidies should be provided from both private and governmental hospitals for the diagnostic tests for those PLHIV who come from remote rural areas, who have below poverty line income and who are marginalised (such as rejected PLHIV from family or community and being an impoverished widow/widower). Allowances should be provided to marginalised PLHIV, similar to the government current policy for pregnant women at the time of delivery. This policy would not only reduce the economic burden of PLHIV, but also encourage them to go for HIV testing, with adherence to the ART medicine. Early diagnosis means early treatment, lesser costs for treatment and loss of productivity.

As the monthly direct costs and productivity losses are very high compared to other diseases, the HIV-affected household faces a considerable economic burden. PLHIV often have limited occupational skills and experience reducing their chances of economic independence. Therefore, we advocate that the Government of Nepal should establish a fuller policy to provide livelihood support (skill development and income generating programmes) for the PLHIV and their family members. The Government of Nepal could provide health insurance to HIV-infected people of as in Merauke, Indonesia.

To implement all of these policies, there would need to be a larger budget for HIV/AIDS. This seems justified from the results of this study showing that HIV/AIDS exerts a greater economic burden on HIV-affected households than other diseases. More focus and support should also be given to those PLHIV who come from remote rural areas, who are below the poverty line income, and who are marginalised due to the disease, and their community reactions.

In order to expand the knowledge of the economic burden experienced by PLHIV, further study of the economic impacts of HIV/AIDS upon the isolated, rejected, widow, widower and separated PLHIV (from family and society due to the disease) and the impact of HIV/AIDS affected households’ children’s education is recommended. In-depth knowledge of these issues would be beneficial in order to formulate a proper policy to enhance the quality of life for such individuals and their families. Likewise, we recommend conducting a longitudinal study for a 6-month period, including a control group to compare the different variables under investigation, involving all types of PLHIV from rural and urban areas.

## Additional file

Additional file 1: Survey Questionnaire. (PDF 350 kb)

## Abbreviations

A: Absenteeism
AIDS: Acquired Immune Deficiency Syndrome
ART: Anti-retroviral therapy
BIIC: Beijing Institute of Information and Control
CD4: Cluster of differentiation 4
DROH: Days recovered by others help
GDP: Gross domestic product
GoN: Government of Nepal
HIV: Human Immunodeficiency Virus
INGO: International Non-Governmental Organisation
LGBT: Lesbian, Gay, Bisexual and Transgender
MoF/N: Ministry of Finance Nepal
MoPH/N: Ministry of Population Health Nepal
NCAIDS: National Centre for AIDS/STD Control and Prevention
NCASC: National Centre for AIDS and STD Control
NGO: Non-Governmental Organisation
NHRC: Nepal Health Research Council
NRs: Nepali Rupees
P: Presenteeism
PLHIV: People living with HIV/AIDS
SLC: School leaving certificate
STD: Sexually Transmitted Disease/s
STI: Sexually transmitted infection
UK: United Kingdom
UNAIDS: Joint United Nations Programme on AIDS
UNDP: United Nations Development Programme
US$: United States Dollar
VAS: Visual analogue scale
VL: Visceral leishmaniasis
