# Supplementary material for: The economic burden of HIV/AIDS on individuals and households in Nepal: a quantitative study
Source: BMC Health Serv Res. 2017 Jan 24;17:76. doi: 10.1186/s12913-017-1976-y (PMC5259845; doi:10.1186/s12913-017-1976-y)
Supplement: Additional file 1: — Survey Questionnaire. (PDF 350 kb) [file 12913_2017_1976_MOESM1_ESM.pdf]

SURVEY QUESTIONNAIRE

1. BACKGROUND INFORMATION			
1.1 Region (RR)			
1.2 District (DD)			
1.3 Treatment site (TS)			
1.4 Household/Respondents ID (RRDDTS---)			
1.5 Date of interview (ddmmyyyy)			
1.6 Time of start of survey		am	pm

(Now I am going to ask you about the personal information which includes about your age, address, occupation etc. but not the name).

2.	PERSONAL INFORMATION		
	**Questions and Filters**	**Coding Categories**	**Filter**
2.1	**In what month and year were you born?**	Month…………<br>Year…………..<br>(if did not get answer go to 2.2)	If got answer go to 2.3
2.2	**How old were you on your last birthday?**	Age (Years) …………	
2.3	**What is the gender of the respondent?**	1. Male<br>2. Female	Only to be asked to the respondent if in doubt
2.4	**How long have you been living continuously in the current address** (name of current address)? If less than one year, write down in month)	**Current Address:**<br>Years………..Month……..<br>Always……..95<br>Visitor………96<br>**Permanent Address:** ……………	

**2.5**	**What is your educational qualification?**	None …………………………01<br>Primary ………………………02<br>Secondary/under SLC ………..03<br>Above SLC/Intermediate …….04<br>Bachelor degree………………05<br>Postgraduate degree …………06<br>Above Postgraduate …………07<br>Other …………………………96	
**2.6**	**What is your main occupation?**	Employment (?)...………………...01<br>Self-employment/business…..........02<br>Farming…………………………..03<br>Housework/childcare…………….04<br>Retired …………………………...05<br>Student …………………………..06<br>Unemployed …………..................07<br>Too ill to work/handicapped……..08<br>Other (specify)………….............96	…………
**2.7**	**What is your surname please?(please choose the caste according to the surname)**<br>Surname…………………..	Brahmin/Chhetri……………..01<br>Newar/Gurung/Thakali……….02<br>Other ethnic groups……….…..03<br>Dalits…………..………………04<br>Other (specify)…………........96	
**2.8**	**Which of the following best describes your sexual orientation?**	Heterosexual………………..01<br>Bisexual …………………....02<br>Gay Man……………………03<br>Lesbian/Gay women……..…04<br>Other (specify)……………...96	

**2.9**	**What is your marital status now?**	Never Married…………….01<br>Married……………………02<br>Living with a partner……...03<br>Widow/widower…………..04<br>Divorced/separated……….05<br>Other (Specify) ……………96	
**2.10**	**When did you diagnose as HIV positive?**	Month ………………..<br>Year …………………..	
**2.11**	**Are you under Antiretroviral Therapy (ART)? If yes, please specify date of start.**	Yes …………………01 (Year:…………Month………….)<br>No ………………….02	
**2.12**	**What is your current CD4 level?**	More than 400/mm^3…………..01<br>200 to 400/mm^3………………02<br>Less than 200mm^3……………03<br>Don't know…………………..04	(if not known, ask ART No.)
**2.13**	**How do you feel about your current health status? Is it normal, medium or poor?**	Good…………………………01<br>Medium…………………… 02<br>Poor……………………… 03	
**2.14**	**Have you got health insurance?**	Yes…………………………01<br>No…………………………..02	

(Now, I am going to speak with you about your household members, their age, education and occupation)

**3. HOUSEHOLD INFORMATION**				
*We would like you to list names of all members of your household who usually **sleep** and **eat** with you (*Total family members staying together…………)				
**SN**	**3.1 Relationship to the respondent**	**3.2 Age at last birthday (Years)**	**3.3 Education (code a)**	**3.4 Occupation (code b)**
**01**				
**02**				
**03**				
**04**				
**05**				
**06**				
**07**				
**08**				
**09**				
**10**				
	**Code a**		**Code b**	
	None …………………………..01<br>Primary ………………………..02<br>Secondary/under SLC ………..03<br>Above SLC/Intermediate …….04<br>Bachelor degree………………05<br>Postgraduate degree …………06<br>Above Postgraduate …………07<br>Other (please specify..………..96		Employment……………………...01<br>Self employment/business…..........02<br>Farming…………………………..03<br>Housework/childcare…………….04<br>Retired …………………………...05<br>Student …………………………..06<br>Unemployed …………..................07<br>Too ill to work/handicapped……..08<br>Other (specify)………….............96	
**3.5**	**Who is the head of the household (relationship)?.............................(male/female)**			

(Now, I want to ask you about your household monthly income and monthly expenditure)

**4.**	**MEASURE OF ECONOMIC STATUS**		
**4.I HOUSEHOLD INCOME**			
**4. I.1**	**What are sources of your income?**	My employment…………………..01<br>My spouse's employment ……….02<br>My son's employment……………03<br>My daughter's employment………04<br>My business………………………05<br>My farming………………………..06<br>Others (specify)…………………...96	 <br>go to 4.I.2<br><br>go to 4.I.3<br>go to 4.I.4<br>go to 4.I.5
**4. I.2**	**How much income do you get from the employment of you and/or your family per month?**	Employee NRs (per month)<br>…………. …………………..<br>……………. ………………..<br>……………. ………………….<br>**Total income (NRs)** ………………	
**4. I.3**	**How much income do you get from your business?**	1) Income from business 1 (per month) NRs …………..<br>2) Income from business 2 (per month) NRs …………...<br>3) Income from business 3 (per month) NRs …………...<br>**Total income (NRs)**…………………	
**4.I.4**	**How much income do you get from your farming?**<br>(convert yearly income to monthly later on)	Source Yearly Income (NRs)<br>……………… ……………..<br>……………… …………….<br>……………….. ……………<br>**Total income (NRs)**…………………	
**4.I.5**	**How much income do you get from other sources of income?**	NRs per month………………………..	
	**Total monthly income (NRs)……………………………………**		
	**(Sum up all the total monthly income and verify with the respondents by asking 'your household total monthly income is approximately NRs………..(sum of above income), is that correct?' If there is big difference, please re-check the above income)**		

(Copy total monthly income from above: NRs…………….)

4.E HOUSEHOLD EXPENDITURE (except HIV treatment)	
**4.E.A. We would like you to tell us how much you or your household spent on the following items in the last month?**	
**EXPENDITURE ITEM**	**AMOUNT SPENT** (NRs)
**4E.1 Food expenditure (Money)**	
4.E.1.1 Expenses for rice	
4.E.1.2 Expenses for vegetables/oils/spices	
4.E.1.3 Expenses for milk	
4.E.1.4 Other food expenses	
**Total Money Expenditure for food (sum of all above)**	………...................
**4E.2 Food expenditure (imputed – consumption from own farm)**	
4.E.2.1 Value of rice	
4.E.2.2 Value of vegetables/oils/spices	
4.E.2.3 Value of milk	
4.E.2.4 Value of other food	
**Total Imputed Expenditure for food (sum of all above)**	……………………….
**4E.3 Expenditure on food (sum of Money & imputed)**	**……………………….**
**4E.4 Non-food expenditure**	
4E.4.1 Education	
4E.4.2 Health (other than direct cost of treatment of HIV in no. 5)	
4E.4.3 Water	
4E.4.4 Lighting	
4E.4.5 Telephone/mobiles	
4E.4.6 House rent	Money cost………..<br>Imputed cost………
4E.4.7 Municipality tax etc.	
4E.4.8 Garbage/refuse collection	
4E.4.9 Transport	
4E.4.10 Toiletries (soap, toilet paper, etc.)	
4E.4.11 Religious	

4E.4.12 Funeral donations/gifts	
4E.4.13 Other non-food expenditure – payments (as wages, etc)	
4E.4.14 Other non-food expenditure (specify) 1………………	
4E.4.15 Other non-food expenditure (specify) 2………………	
4E.5 Remittances sent out	
**4.E Total Households Expenditures (NRs)**	**………….**
**(Sum up all the total monetary cost and verify with the respondents by asking 'your household total monthly expenditure is approximately NRs…………(sum of above expenditure), is that correct?' If there is big difference, please re-check the above major expenses)**	

(Compare the total monthly income with the total monthly expenditure. If the expenses are considerably higher than the income, the household must use savings or sale assets or borrowed or took loans from others)

(Now, I want to know about the cost of treatment for you. It includes travel cost, medicine, diagnostic costs and cost for accompanying persons as well. I also ask how much time you and your accompanying person spent for the treatment).

**5. DIRECT COSTS FOR HIV/AIDS TREATMENT**			
**5.1 What was the direct cost incurred for your last visit to the treatment centre?**			
**COST OF ILLNESS (at the last visit)**			
**A. Cost of treatment (ask respondents if they have got receipts)**		**Items**	**Costs(NRs)**
		**Doctor's fees**	
		**Diagnostic costs**	
		**Medicine cost**	
		**Other medical costs (1) (specify)…………**	
		**Other medical costs (2) (specify) ....**	
		**Total medical costs (NRs)**	**…………**
**B. Cost of accessing treatment centre**		**Items**	
		**Travel cost**	
		**Food cost**	
		**Lodging cost**	
		**Other costs (1) (specify)………………….**	
		**Other costs (2) (specify)………………….**	
		**Total accessing costs (NRs)**	**…………**
		**Total costs (NRs)**	**…………**
**5.2**	**How much time did you lose to access the treatment centre for your last visit?**	**On the way:**<br>**Hours………**<br>**Days ………..**<br>**In the treatment centre:**<br>**Hours………**<br>**Days ………..**	
**5.3**	**Did you take an accompanying person with you in that visit?**	**Yes……….01 (go to 5.4)**<br>**No………..02 (go to 6)**	

**5.4**	**How much cost did you pay for accompanying person for the following items?**	**<u>Items</u>** **<u>Costs (NRs)</u>**<br>**Travel …………………**<br>**Food…………………...**<br>**Lodging ……………….**<br>**Other costs (specify) …**<br>**Total expenses (NRs)…**	
**5.5**	**How much time did your accompanying person lose to access the treatment centre for your last visit?**	**Hours………**<br>**Days ………..**	

(Now, I am going to speak about your normal daily activities at the time you were not ill - what do you do in a normal day)

**6. RESPONDENT'S NORMAL DAILY ACTIVITIES**	
**6.1**	**In a normal working day, how many hours do you sleep normally?**
**6.2**	**Beginning from the time you wake up, please tell us about the activities that you undertake on a normal working day. (explain about time guide to the respondents to keep track of time)**

**Activities**	**How much time do you spend on each activity?**
	**Hours and minutes**
Morning fresh-up activities (bath, shower, brush, toilet etc.)	
Daily business work (work in own shop)	
Daily agriculture work (work in own farm)	
Normal household work (cooking, dish washing, fetching water, cleaning house etc)	
Job/office work (work in the office or work)	
Wage labour (working for daily wages as labour)	
Child care work (caring children, feeding, bathing etc.)	
Daily entertainment/rest (watching TV, doing rest, chatting with friend etc.)	
Daily travel/walk to job/ wage labour/ farming/business	
Daily travel/walk for fitness	

(Sum up the total working hours with the sleeping hours, the total hours should not exceed 24 hrs)

(Now, I am going to discuss about your productivity loss due to the disease)

**7. PRODUCTIVITY COSTS**			
**7.1**	**Was there any times when you been completely unable to carry out your normal daily activities in the last two month period?**	Yes…….01<br>No…..…02	(go to7.2)<br>(go to 7.3)
**7.2**	**For how long were you completely unable to attend your normal daily activities in the last two month (60 days) period?**	Days ……...	
**7.3**	**For how long have you worked in a state of poor health in the last two month (60 days) period?**	Days ……...	
**7.4**	**If these blocks [present 5 blocks] represent the work you normally did in a state of good health, can you <u>pick the number that represents your work efficiency when you were ill</u>?** (Enter number of blocks picked)		

0	1	2	3	4	5
Illness did not permit me to work					Illness did not affect my work

(Use separate card to facilitate the respondents to pick the correct block)

(Check: answer of question 7.4 never would be block 0 and 5)

(Now, I want to speak with you about the coping strategies you or your family used for the treatment of the disease and to cover work loss due to illness).

**8.**	**COPING STRATEGIES**		
**8.1**	**How did you or your family manage costs for treatment and days lost due to illness?**	By using cash (income +saving)01<br>Sale of assets…………………..02<br>Loans ………………………....03<br>Borrowing…………………..…04<br>Wage labour………………..…05<br>Gifts …………….…………......06<br>Insurance……..…………..…...07<br>Children taken out of school….08<br>Others (specify, with value).....96	(go to 8.2)<br>(go to 8.3)<br>(go to 8.4)<br>(go to 8.5)<br>(go to 8.6)<br>(go to 8.7)
**8.2**	**If using income or savings, please tell me how much money you spent for your treatment?**	NRs…………………..	
**8.3**	**If sale of assets, please specify what has/have been sold and how much money was received?**	**Items** **Value (NRs)**<br>House…………………<br>Land ………………….<br>Jewellery ……………..<br><br>**Livestock and Poultry**<br>…………………………<br>…………………………<br>…………………………<br><br>**Household Equipments**<br>………….…………….<br>…...……………………<br>…..…………………….<br>………………………..<br>………….…………….<br>Other (specify)………..<br>**Total Value (NRs**)………	

**8.4**	**If taking loan, what is the source of the loan, how much money was received, interest rate, and payback period?**	**Source** **Loan (NRs)** **Interest rate (%)** **Payback Period**<br>Relatives………………………………………<br>Friends/ neighbour..…………………………..<br>Local saving group……………………………<br>Government banks or cooperatives…………..<br>Private banks …………………………………<br>Other source (specify)………………………..	
**8.5**	**If borrowed, please tell me the source, amount of borrowing and payback period?**	Source …………………….<br>Amount (NRs)……………..<br>Payback period…………….	
**8.6**	**If you did wage labour, who did and how much did you earn?**	Who did labour……………<br>Money earned (NRs)………………………	
**8.7**	**If you received gifts, from whom and how much money was received?**	**Items** **Value (NRs**<br>Relatives……………………<br>Friends/neighbour …………<br>Other (specify) ……………	
**8.8**	**If you used insurance, from where and how much was received?**	**Insurer's Name** **Amount received (NRs)**<br>…………… ……….<br>…………… ……….<br>………….. ………..	
**8.9**	**If children were taken from school, why did you take children out of school?**	Cannot pay tuition fee………………..01<br>Need children to carryout household activities……………………………...02<br>For wage labour work……..…………03<br>For other purpose (specify)…………...96	
**8.10**	**If other sources were used for coping, how much money was used from other source?**	NRs…………………	

**8.11**	**Did anybody help you to carry out your normal daily activities when you were ill?**	Yes…………..1 (go to 8.12)<br>No……………2 (go to 9.0)	
**8.12**	**If you received help, who assisted, for how many days, and for what type of work you received assistance for your normal activities during the time of your illness?**<br>**<u>Who (write down relation to the respondent)</u>** **<u>days for caring</u>** **<u>days for daily activities</u>**<br>………………………….. ………………… ………………<br>…………………………… …………………. ………………<br>…………………………… …………………. ………………<br>…………………………... …………………. ..……………<br>…………………………... ………………… ………………		
	**<u>Code</u>**<br>Other family members…01 (example- 1.1 sister, 1.2 brother, 1.3 mum, 1.4 dad etc.)<br>Relatives……………….02 (example- 2.1 mother- in- law, 2.2 Father-in-law etc.)<br>Neighbours/friends….…03 (example- 3.1 neighbour, 3.2 friend etc.)<br>Hired someone……… 04 (go to 8.13)<br>Other (specify) ………96		
**8.13**	**If hired labour, how much did you pay them?** (If payment was in kind ask for value, and only fill if applicable)	Daily wage rate (NRs)……….<br>Total wages (NRs)..................(wage rate x total days)<br>Value of the kind (if applicable) (NRs)…….	
**8.14**	**If these blocks [present 5 blocks] represent the work that you normally do in a state of good health, can you <u>pick the number that represents the amount of work done by the persons who assisted you</u>?** (Enter number of blocks picked)		

0	1	2	3	4	5
No one assisted me					Assistance fully covered all my activities

(Use separate card to facilitate the respondents to pick up correct block)

(If the people helped you fully substituted your work (block 4 or 5), did those who assisted you have to give up some their normal work? ……………………………

(It is the last section of the questionnaire, now I want to ask you about the stigma and discrimination associated with the disease)

9. STIGMA AND DISCRIMINATION

**9.1**	**Could you tell me please who knows about your HIV status?**	Family members (who………. …)..01<br>Other relatives (who……..........)..02<br>Neighbour/ friends ………………...03<br>Employer…………………….…….04<br>Health professional ……………..…05<br>Other (specify) ……………………96	
**9.2**	**Did you feel discrimination from anybody after diagnosis of the disease?**	Yes……………………………….....01<br>No……………………………….....02	 (If no end the Survey)
**9.3**	**If you feel discrimination, which of the following showed such behaviour towards you?**	Family members ……………………01<br>Other relatives ……………………....02<br>Neighbour/ friends ……………….....03<br>Employer…………………….…..….04<br>Health professional …………………05<br>Other (specify) ………………………96	(go to 9.4)<br>(go to 9.5)<br>(go to 9.6)<br>(go to 9.7)<br>(go to 9.8)<br>(go to 9.9)
**9.4**	**If you felt discrimination from your family, please mention how they behave towards you.**	Don't visit me……………………….01<br>Don't touch me…………………..…02<br>Don't eat with me……………….….03<br>Don't sit with me………………….....04<br>Abuse me……………………….......05<br>Beat me…………..........................06<br>Hide me so no one knows I have HIV…………………………………07<br>Other (specify)……………………96	
**9.5**	**If you felt discrimination from other relatives? Please mention how they behave towards you.**	Don't visit me…………………...…01<br>Don't touch me…………………......02<br>Don't eat with me……………….……..03<br>Don't sit with me…………………..….04<br>Abuse me……………………………05<br>Other (specify)……………………....96	

**9.6**	**If you felt discrimination from neighbours or friends? Please mention how they behave towards you.**	Don't visit me……………………….....01<br>Don't touch me……………………..…..02<br>Don't eat with me…………………...03<br>Don't sit with me…………………...04<br>Abuse me..05<br>Family was excluded from community events…………………………….….06<br>Neighbours stopped visiting the house………………………………...07<br>Was asked to leave the community…...08<br>Children were discriminated against in school……….…………….........09<br>Stopped give and take ……………..10<br>Stopped labour exchange................11<br>Other (specify)……………………...96	
9.7	If you felt discrimination from employer/s? Please mention how they behave towards you.	Removed from job……….............01<br>Given less wages/salary..…………....02<br>Did not give my wages/salary ………03<br>Other (specify)…………….…….…96	
9.8	If you felt discrimination from health professional/s? Please mention how they behave towards you.	Refused to treat you/denied access to medical treatment or care…………...01<br>Experienced a delay in the provision of health services/treatment…………….02<br>Verbally abused…………………..…03<br>Other (specify)………………………96	
9.9	If you feel discrimination from others, please which mention how they behave towards you?	………………………………<br>………………………………<br>………………………………	
9.10	Did you feel other types of discrimination which affect your economic capacity? Explain.	……………………………………………<br>……………………………………………<br>……………………………………………<br>……………………………………………<br>…………	

THANK YOU!!!

**Time interview ended**										**Am**	**pm**
